# Development and validation of a novel fluorometric approach utilizing flow injection analysis for the measurement of vilazodone: application to dosage form and spiked human plasma

**DOI:** 10.1186/s13065-025-01458-6

**Published:** 2025-04-13

**Authors:** Al Amir S. Zaafan, Sayed M. Derayea, Mohamed Oraby, Dalia M. Nagy

**Affiliations:** 1 Department of Pharmaceutical Analytical Chemistry, Faculty of Pharmacy, Sohag University, Sohag, 82524 Egypt; 2 Analytical Chemistry Department, Faculty of Pharmacy, Minia University, Minia, 61519 Egypt; 3 Pharmaceutical Chemistry Department, College of Pharmacy, Al-Esraa University, Baghdad, Iraq

**Keywords:** Vilazodone, Flow injection, Fluorescence, Dosage form, Spiked human plasma

## Abstract

A direct, precise, rapid and simple flow injection approach has been applied to determine vilazodone HCl (VZN) in pharmaceutical dosage forms and biological fluids. VZN has an indole ring as part of its structure, which gives it a significant native fluorescence. The study was based on determining the strong intrinsic fluorescence of VZN, which was measured at 486 nm after excitation at 241 nm. Phosphate buffer (pH 5, 10 mM): Acetonitrile (40:60, v/v) was utilized as the carrier solution, with a flow rate of 0.5 mL min^− 1^. Based on peak area, the calibration graph was linear over a concentration range of 10–300 ng mL^− 1^ of VZN with a correlation coefficient (r) of 0.9999. The quantitation limit was 9.62 ng mL^− 1^, while the detection limit was 3.17 ng mL^− 1^. Moreover, the suggested method was used to accurately measure VZN in its tablet dosage form. Additionally, the studied drug was also satisfactorily measured in blood using the suggested flow injection methodology. The approach was validated according to ICH specifications.

## Introduction

**Fig. 1:**
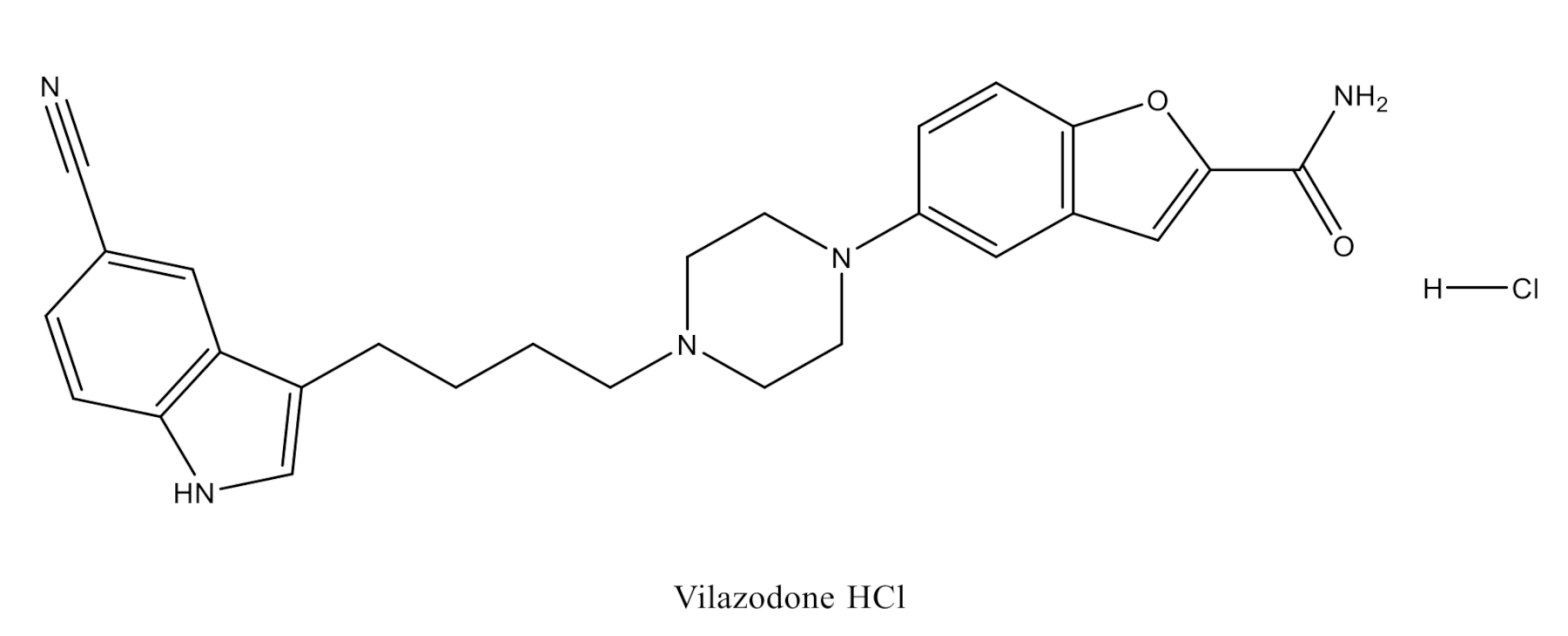
The chemical structure of VZN

Vilazodone HCl (VZN, Fig. 1) is known chemically as 5-(4-[4-(5-cyano-1 H-indol-3-yl)butyl]piperazin-1-yl)benzofuran-2-carboxamide hydrochloride [1]. It comprises indole-piperazine, which makes use of its properties as a partial agonist of the 5-HT_1A_ receptor and a selective serotonin reuptake inhibitor (SSRI). This inhibitor of serotonin reuptake has a strong affinity for D2 receptors. It also has an impact on many serotonin receptor subtypes [2]. VZN is the first drug in a new class of antidepressant drugs that combines the strength of SSRIs with partial agonistic effect on the 5-HT_1A_ receptor. The FDA authorized it for use as a therapy for major depressive disorder (MDD) in adult patients in January 2011. By inhibiting serotonin transporter, desensitizing its receptors, and ultimately boosting its neurotransmission as well, vilazodone works similarly to SSRIs [3]. Its antidepressant properties result from a combination of its effect as a SSRI and its partial agonistic activity on the 5-HT_1A_ receptor. According to reports, VZN’s absolute bioavailability is 72% in fed conditions. It has also been demonstrated that food elevates its bioavailability; therefore, taking it with food might be advantageous [4]. VZN is well distributed, metabolized primarily in the liver, and approximately one to two% of the administered dosage is recovered in intact form in a person’s urine and faeces [5]. CYP450 3A4 coenzyme in the liver is the enzyme involved in the metabolism of VZN. So, the dose of VZN should be diminished when strong inhibitors of the 3A4 enzyme are used simultaneously. Almost all of the beneficial effects of vilazodone are attributed to its main substance; no biologically active metabolites have been identified yet [6].

According to a survey of the literature, only a few analytical approaches have been used to determine VZN in biological fluids and pharmaceutical formulations, including spectrophotometry [2, 7–9], spectrofluorimetry [10, 11], HPTLC [8, 12], HPLC [1, 13–16], LC/MS [3]^,^ and UPLC methods [4, 5]. Spectrophotometric methods are rarely employed for biological fluid analysis due to their lower sensitivity [17]. Despite being highly sensitive and appropriate for investigating drug substance degradation and pharmacokinetics, methods involving chromatography are limited by their lengthy process, high equipment costs, large quantities of highly pure organic solvents, and staff training requirements [18, 19]. The consumption of excessive volumes of organic solvents in HPLC not only increases the cost of the analysis but also has a bad influence on the environment. Furthermore, the cost of instrumentation would be extremely high if mass spectrometry was used for detection [3–5, 14]. In addition, analysis with HPLC or UPLC requires a time-consuming and laborious sample pretreatment step, which has a bad impact on time efficiency. A key area of study in modern analytical chemistry, flow analysis has acquired importance due to the wide-ranging development of specialized instruments, the numerous positive aspects it offers over conventional techniques, a wide variety of scientific and obvious papers, and many types of applications that are adjusted to meet the needs of many different domains, including ecological and clinical chemistry, nutrition and agricultural chemistry, and chemical research [20, 21]. On the other hand, flow injection analysis (FIA) offers several key advantages that enhance its utility in analytical chemistry. Firstly, it enables rapid sample processing, and samples do not need tedious prior treatment, allowing for high-throughput analysis with minimal sample volumes. FIA also provides excellent precision and accuracy, as that obtained with HPLC. Additionally, the technique’s adaptability allows it to be used with a wide range of reagents that could not be used with HPLC or UPLC, facilitating diverse applications across various fields.

Techniques utilizing spectrofluorimetric detectors require only minimal preparation of samples, have a great sensitivity, and are particularly selective because they have two spectra [22–24]. The method validation complies with the guidelines provided by ICH [25]. The current work is innovative since, as of yet, no published flow injection method for determining VZN has been identified. We were motivated by this to develop a novel flow injection-based technique with fluorescence detection for VZN determination. In light of this, the sole goal of this research is the development of a flow injection-fluorometric approach that is highly sensitive, quick, and consumes small amounts of solvents for measuring VZN in body fluids and pharmaceutical forms.

## Experimental

### Instrumentation

The flow injection manifold consisted of a Sykam S 1130 HPLC quaternary pump with an optional integrated online vacuum degasser (Sykam GmbH, Gewerbering, Germany). Hamilton HPLC syringe (Franklin, MA, U.S.A.) was used to inject samples. A detector (RF-20 A, Shimadzu, Kyoto, Japan) attached to the HPLC system was used to perform the fluorometric measurements. In the present technique, the column of the HPLC system was not used. The pH was adjusted with a Jenway 3510 pH meter (Staffordshire, UK). Weighing was done using a Mettler Toledo 5-digit balance (Greifensee, Switzerland).

### Chemicals and materials

VZN was kindly donated by RAMEDA Co. for Pharmaceutical Industries (6th of October City, Egypt). Vilaphoria^®^ 20 mg tablets were obtained from the Egyptian market. Every solvent was HPLC grade and purchased from Sigma Aldrich (Germany). O-phosphoric acid and dipotassium hydrogen phosphate were brought from El Nasr Chemical Co. (Cairo, Egypt).

### Flow injection conditions

The experiment was conducted with a carrier solvent combination of phosphate buffer (pH 5, 10 mM) and acetonitrile (40:60, v/v), which had been filtrated and degassed prior to use, at a rate of 0.5 mL min^− 1^, and fluorescence detection at 486 nm after the excitation wavelength had been set at 241 nm. A 20 µL volume was employed as injection volume, and the study was conducted at ambient temperature.

### Preparation of carrier solvent

Add about 0.871 g of dipotassium hydrogen phosphate, dissolve it in the solution, and mix vigorously in a flask with a volume of 500 mL with 500 mL of water that has been double-distilled. The pH of the resultant solution should then be adjusted to 5 by adding ortho phosphoric acid. To get rid of gas, the solution needs to be filtered using a 0.22 μm membrane filter. It is subsequently mixed with acetonitrile (40: 60, V/V).

### Preparation of standard solution

A stock solution of 1 mg mL^− 1^ was prepared by properly weighing 10 mg of authentic VZN powder and dissolving it with 10 mL of methanol in a volumetric flask (10 mL). Utilizing the carrier solvent, the working standard solution of VZN was obtained. In the refrigerator, stocks were reserved. Dilution with the same solvent was used to produce solutions of 5, 10, 20, 50, 100, 150, 200, 250, and 300 ng mL^− 1^ VZN for calibration curve construction.

### Sample preparation

Ten Vilaphoria 20 mg tablets were carefully inserted into a mortar, and then the powder was obtained by grinding the tablets until very fine. The drug was then extracted by dissolving the powder in HPLC-grade methanol in a volumetric flask (50 mL) through sonication for about 30 min using a tablet powder containing 20 mg of VZN that was obtained from there. Whatman filter paper grade No. 1 was then utilized to filter the final solution, and the first portion was discarded. Dilution with the carrier solvent was used to produce the working solution. Five determinations of the same VZN concentration were compared using an accepted assay method. Using either the relevant regression equation or the calibration graph, precisely how much of the tablets was determined.

### Spiked human plasma preparation

In order to investigate the proposed approach’s practical use, the viability of using it for real-life sample analysis had to be evaluated, specifically determining precisely how much VZN is present in human plasma samples. About 100 µL of various VZN concentrations were dissolved in carrier solvent, spiked with 100 µL of free drug human plasma, and 200 µL of acetonitrile was added for protein precipitation. The mixture was then vortexed for 30 s and centrifuged at 4000 rpm for 30 min to achieve a final concentration of VZN within the linear range. After that, the produced supernatant was subjected to the analysis procedure [26]. This extensive procedure attempted to verify that the established approach could be used for practical applications, specifically in terms of measuring VZN concentrations in human plasma samples using precise and successive analytical procedures. Three identical samples of each concentration were used in the experiment, and a blank experiment was conducted alongside.

## Results and discussion

**Fig. 2:**
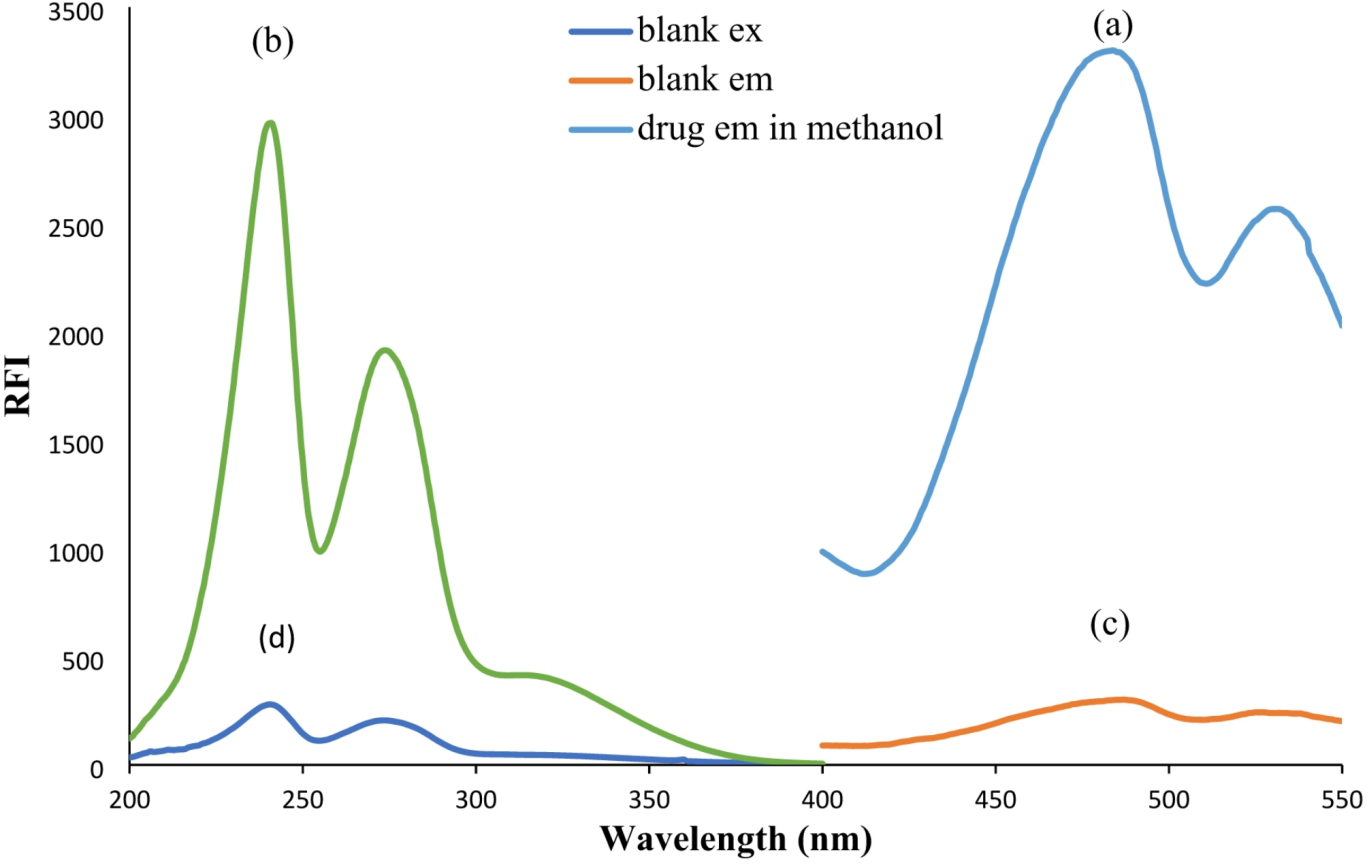
Emission (**a**) and excitation (**b**) spectra of 100 ng mL^-1^ VZN, and emission (**c**) and excitation (**d**) spectra of blank all in methanol

The chemical structure of VZN illustrated in Fig. 1 shows two highly conjugated systems with planar configurations, indole and benzofuran moieties. Thus, VZN is expected to exhibit intrinsic fluorescence (Fig. 2). However, low fluorescent activity was observed for VZN under alkaline conditions. This abnormal emission behavior could be attributed to the intramolecular photoinduced electron transfer (PET) by the free lone pair of electrons of the nitrogen atom of the piperazinyl ring. The optimal fluorescence of the drug was achieved only in an acidic environment where the drug was protonated and thus exhibited more fluorescence emission than the free drug. Protonation of the nitrogen atom of the piperazine ring in the presence of phosphate buffer (pH 5.0) could effectively result in turning off the PET process. This renders the π electrons of the aromatic system in VZN to be fully available for π to π* transitions, and consequently results in enhanced fluorescence emission [27, 28].

PET is an established mechanism involved in the fluorescence quenching phenomenon. It is a process where an electron is transferred from a PET donor to an excited acceptor and results in a reduction of the fluorescence of the acceptor. If the energy level of the highest occupied molecular orbital (HOMO) of the donor is high enough, electron transfer to the excited acceptor is favored, and the result would be low fluorescence emission or quantum yield. Therefore, for fluorescent molecules with strong emission responses, an electron donor with comparatively lower HOMO energy levels is required [29, 30]. Therefore, the HOMO and LUMO energy levels were calculated for both unprotonated and protonated VZN using B3LYP/6-31G methodology. The respective HOMO and LUMO energy levels were calculated as -7.895 eV and − 5.067 eV for bare VZN, while − 8.162 eV and − 5.075 eV were the respective values for the protonated form. These findings suggest that the energy gap between the HOMO and LUMO of the protonated form is smaller than that of the unprotonated form by -3.087 eV and − 2.828 eV, respectively, confirming that the protonation (pH 5.0) significantly enhances the fluorescence emission of VZN.

**Fig. 3:**
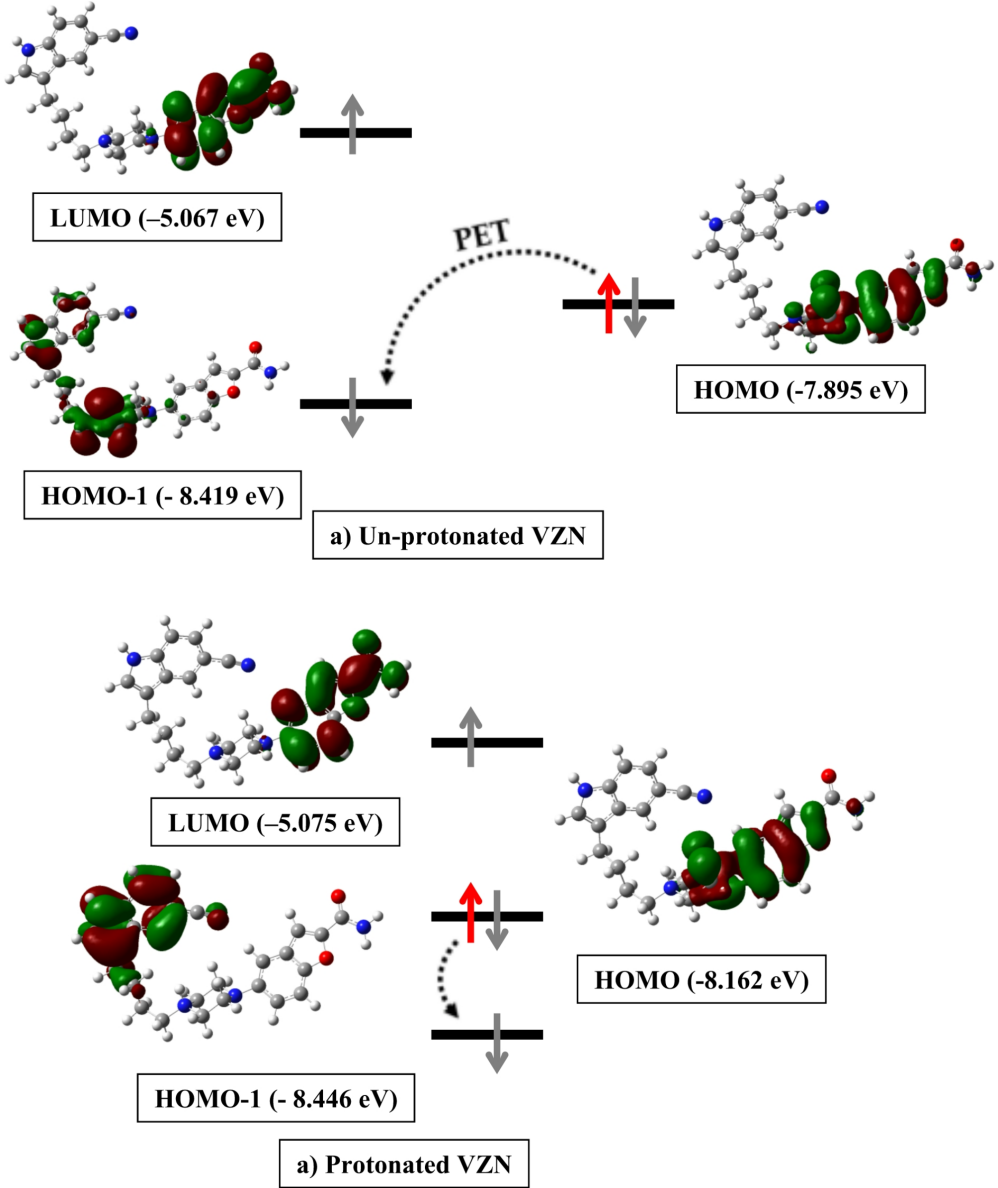
HOMO, LUMO and HOMO-1 molecular orbitals for VZN and its protonated form

Furthermore, LUMO, HOMO, and HOMO-1 distributions for both the bare and the protonated drug are generated and are shown in Fig. 3. LUMO is primarily distributed over the benzofuran moiety in both the protonated and free VZN which indicates that benzofuran is the electron acceptor moiety in both forms, which is consistent with the expected behavior of such systems, where the LUMO is often involved in electron acceptor properties. While, HOMO is distributed over both indole and piperazinyl moieties, also for both free and protonated drug. On the other hand, there is a great difference in the distribution of HOMO-1 over the protonated and unprotonated forms. In the case of the protonated drug, the HOMO-1 is mainly distributed over the indole ring, while it is moderately distributed over the indole, slightly over the aliphatic carbon chain, and predominantly distributed over the piperazine ring. In the case of the unprotonated drug, the significant distribution of HOMO-1 over the piperazine suggests that it is able to donate an electron to the LUMO of a nearby acceptor. Such an electron transfer can lead to a non-radiative decay pathway, resulting in fluorescence quenching. In the protonated state of VZN, the HOMO-1 is mainly localized on the indole ring. This distribution indicates that the protonated form of VZN has a lower tendency to undergo PET since the electron-donating capability of the piperazine decreases. Thus, if the PET process is hindered, the fluorescence can be recovered or increased [31, 32].

### Optimization of experimental parameters

#### Selection of detection wavelength

VZN solution (10 µg mL^− 1^) was prepared by correctly diluting the standard solution in order to choose an analytical wavelength. Ten mg of VZN powder was transferred to a 10 mL volumetric flask, dissolved in methanol, and adjusted to the final volume. From this solution, 0.1 mL is pipetted into a 10 mL volumetric flask and completed to the mark with methanol to give a concentration of 10 µg mL^− 1^, and then it was scanned at 200 to 400 nm by a double-beam spectrophotometer using methanol as a blank. The maximum wavelength of VZN was determined from the spectrum to be 241 nm; therefore, that wavelength was used as the excitation wavelength for the investigation. After that, 0.5 µg mL^− 1^ of VZN solution was achieved by proper dilution using the same solvent, and the emission wavelength was measured using a spectrofluorometer after excitation at 241 nm. As a result, 486 nm was chosen as the analysis’ emission wavelength because it was found that VZN exhibits its strongest fluorescence at this wavelength (Fig. 2).

#### Selection of carrier solvent and pH

Following a number of initial trials using various combinations and ratios of methanol, water, ACN, and buffers, the strongest fluorescence was obtained when buffer was mixed with acetonitrile. In order to determine the optimum ratio of the two components to be used in this approach, different ratios have been tried to obtain the maximum fluorescence, including (20:80, 40:60, 50:50, 70:30, 80:20, and 90:10). Phosphate buffer (10 mM): acetonitrile (40:60 v/v) was found to be the optimum carrier solvent. Different pH ranges were then tried to obtain the optimum pH, and it was obtained that pH 5 gives the maximum intensity of fluorescence.

#### Flow rate optimization

The flow rate of the FIA system has a significant influence, as it could affect the area under the peak, which represents fluorescence response. For optimization of the flow rate, different flow rates were selected to be tried (0.5, 0.8, 1, and 1.2 mL/min), and it has been found that when the flow rate was increased towards 1.2, the peak area was significantly decreased so that 0.5 mL/min was selected as the optimum flow rate, which gives the highest peak area and improves peak resolution.

### Validation of the proposed method

According to ICH guidelines [25]^,^ the established analytical methodology was validated, and the results were represented as percentages.

#### Linearity

**Fig. 4:**
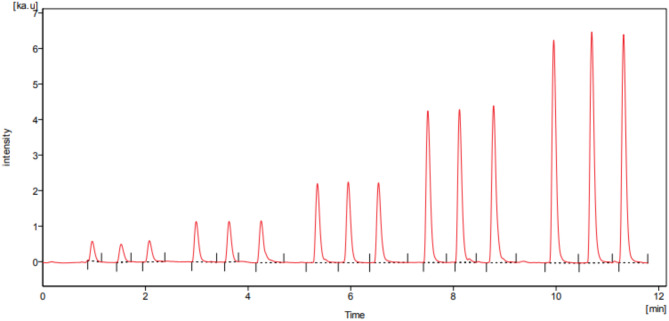
FIAgram of VZN 10, 50, 100, 200, 300 ng mL^-1^

Several standard VZN solutions that were being investigated at different concentrations have been subjected to the general analytical method. The FIAgram of VZN is demonstrated in Fig. 4. The calibration curve of VZN is obtained by plotting the peak area versus the final concentration of the drug. The suggested assay’s linearity graph for the concentration range of 10–300 ng mL^− 1^ was obtained, and its correlation coefficient was 0.9999. The values for the calibration data, regression data, and correlation coefficients are presented in Table 1.

**Table 1:** Regression equation and validation parameters for the proposed method

Parameters	Vilazodone
Linear range (ng mL^− 1^)	10–300
Standard deviation of slope (S_b_)	0.69
Intercept (a)	2272.56
Standard deviation of intercept (S_a_)	68.51
Correlation coefficient (r)	0.9999
Determination coefficient (r^2^)	0.9998
Standard deviation of slope (S_b_)	0.69
Number of determinations	5
LOD (ng mL^− 1^)	3.17
LOQ (ng mL^− 1^)	9.62

#### Limit of detection (LOD) and limit of quantitation (LOQ)

Although it might not be quantifiable in the specific circumstances of the experiment, LOD is the least amount in a sample that can be detected. While LOQ refers to the smallest amount of analyte in a sample that can be quantified with adequate accuracy and precision. Detection and quantitation limits were calculated by means of the subsequent formula LOD = 3.3(SD)/S and LOQ = 10(SD)/S, where SD = the standard deviation of intercept and S = the slope of the calibration curve. The values of LOD and LOQ are demonstrated in (Table 1).

#### Accuracy

The recovery of VZN had been calculated using the general procedure while evaluating the accuracy of the proposed method. The recovery evaluations were performed in three replicates. The standard deviations were below 2.0, and the percent recoveries ranged from 98 to 102%, showing that the accuracy of the process under assessment was sufficient. The outcomes are shown in (Table 2).

**Table 2:** Evaluation of the accuracy of the analytical procedure for the determination of VZN (*n* = 3)

Amount taken (ng mL^− 1^)	Amount found (ng mL^− 1^)	Recovery͙͙ (%)	Standard deviation	RSD (%)
20	19.93	99.64	1.80	1.80
50	49.52	99.04	0.78	0.79
100	100.53	100.53	1.08	1.08
200	196.76	98.38	1.56	1.59
300	301.59	100.81	0.63	0.62

RSD = Relative standard deviation

#### Precision

Through measuring corresponding responses three times during the same day and three successive days for intra-day and inter-day precision, respectively, at concentrations of 50, 200, and 300 ng mL^− 1^, an intra-day and inter-day precision investigation of VZN has been assessed. A percent relative standard deviation (%RSD) of no more than 2.0% was determined to be acceptable, with calculated recovery values ranging from 98 to 102%. (Table 3) shows the precision results for both intra-day and inter-day.

**Table 3:** Evaluation of the precision of the proposed flow injection method for the determination of VZN

Precision level	Added concentration (ng mL^-1^)	Measured concentration (ng mL^-1^)	Recovery* (%)	Standard deviation	RSD (%)
Intra-day (Day1, *n* = 3)	50	49.6	99.20	0.71	0.72
	200	197.90	98.95	1.15	1.16
	300	301.20	100.40	1.40	1.39
Inter-day (Whole days, *n* = 9)	50	49.42	98.83	1.88	1.90
	200	198.70	99.35	1.67	1.69
	300	301.77	100.59	0.85	0.84

* Number of determinations = 3

RSD = Relative standard deviation

#### Robustness

By analyzing VZN under slightly different experimental conditions, the robustness was assessed, such as carrier solvent composition (± 2%), slight flow rate fluctuations (± 0.1 mL/min), pH (± 0.1), and detection wavelength (± 2 nm), and the results are represented in (Table 4). Minor variations in any of the investigated variables had no major impact on the outcomes of the approach because the calculated SD was less than 2%. This demonstrates the robustness of the suggested approach.

**Table 4:** Robustness study of the proposed method for the determination of 200 ng mL^-1^ VZN (*n* = 3)

Parameter			Recovery͙͙ (%)	Standard deviation	RSD (%)
Optimum condition			98.95	1.15	1.16
Flow rate		0.4	101.51	0.92	0.91
		0.6	99.83	1.34	1.34
Percentage of acetonitrile (%)		58	98.72	1.44	1.45
		62	100.59	0.74	0.74
pH		4.9	100.68	1.38	1.37
		5.1	101.59	0.76	0.75
λ excitation (nm)		239	99.09	0.84	0.85
		243	99.25	0.94	0.95
λ emission (nm)		484	99.55	1.07	1.08
		488	98.48	1.52	1.55

RSD = Relative standard deviation

### Application

#### Application to pharmaceutical formulations

The medication content of a commercial dosage form (Vilaphoria^®^ 20 mg tablets) was assessed via the usual analytical methodology. The approach’s predicted percentage recovery of 100.93 + 0.83 was satisfactory. The high recovery rate confirmed that there was no substantial impact from the additives in the tablet. A published approach was also used to evaluate the same product [2]. Statistics were utilized to compare the precision and accuracy of the findings produced using the suggested technique to those acquired using the specified methodology. (Table 5) illustrates that there was no substantial difference between the results of the two methods since the estimated t-test and F-test values did not exceed the tabulated values at a 95% confidence level.

**Table 5:** Analysis of Vilazodone in dosage form (Vilaphoria 20 mg/ tablet) by reported and proposed methods

Parameters	Proposed method	Reported method
% Recovery ^a^	100.93	99.59
Standard deviation	0.83	1.22
Variance	0.86	1.48
Number of measurements	5	5
t-test ^b^	2.05	--
F-test ^b^	2.14	--

^a^ Mean of five determinations

^b^ Tabulated value at 95% confidence limit, F = 6.388 and t = 2.306

#### Spiked human plasma application

The very sensitive FIA approach enabled the analysis of VZN in spiked human plasma. The reported C_max_ value for VZN after administration of a single oral dose was 156 ng mL^− 1^ [33]. Samples of human plasma were mixed with various concentrations of the drug, and acetonitrile was added for protein precipitation. After centrifugation, the proposed analytical procedure was applied. VZN has demonstrated very good recoveries, and there is no significant interference of plasma components that has been observed. Therefore, we aimed to investigate its long-term viability for wider applications in the near future, considering analyzing real biological specimens. The matching medication concentrations were calculated using the linear regression equations, as shown in (Table 6).

**Table 6:** The application of the developed method to measure the drug in spiked human plasma

Drug conc. (ng mL^− 1^)	% Recovery ^a^	SD	%RSD
20	101.41	2.28	2.24
50	99.10	2.06	2.08
100	100.55	1.64	1.63
200	100.51	1.99	1.98

* Mean of three determinations, SD, standard deviation’ RSD: Relative standard deviation

## Conclusion

A novel flow injection-fluorometric methodology was created for this study’s evaluation of VZN and was not previously used for analysis. The suggested methodology is simple to use, accurate, and precise, and it may be used to analyze VZN in its pure form, pharmaceutical dose form, and biological fluids. VZN produced a strong peak when the carrier solution composition of 10 mM phosphate buffer (pH 5): acetonitrile (40: 60, v/v) was adjusted. Over the range of 10–300 ng mL^− 1^, the calibration curve for VZN was found to be linear. The regression equation was found to be Y = 143.55X + 2272.56 with the correlation coefficient *r* = 0.9999, which indicates this method has respectable linearity. These findings illustrate the method’s accuracy, precision, and extreme sensitivity. FIA technique’s numerous rapid and widespread applications in quantitative chemical analysis are mostly due to the analysis’s quick turnaround time and minimal reagent usage. Additionally, because of its high sensitivity, it can be used to analyze biological samples and determine analytes at low concentrations.

## Data Availability

The datasets used and/or analysed during the current study are available from the corresponding author on reasonable request.
